# Stigmatized individuals: a case for precision ethics

**DOI:** 10.47626/2237-6089-2021-0354

**Published:** 2023-03-07

**Authors:** Devon Watts, Jeff D’Souza, Marco Antonio Azevedo, Gary Chaimowitz, Flavio Kapczinski

**Affiliations:** 1 St. Joseph’s Healthcare Hamilton Hamilton ON Canada St. Joseph’s Healthcare Hamilton, Hamilton, ON, Canada.; 2 Neuroscience Graduate Program McMaster University Hamilton ON Canada Neuroscience Graduate Program, McMaster University, Hamilton, ON, Canada.; 3 Institute on Ethics & Policy for Innovation McMaster University Hamilton ON Canada Institute on Ethics & Policy for Innovation, McMaster University, Hamilton, ON, Canada.; 4 Escola de Humanidades Universidade do Vale do Rio dos Sinos São Leopoldo RS Brazil Escola de Humanidades, Universidade do Vale do Rio dos Sinos, São Leopoldo, RS, Brazil.; 5 Instituto Nacional de Ciência e Tecnologia Translacional em Medicina Porto Alegre Brazil Instituto Nacional de Ciência e Tecnologia Translacional em Medicina (INCT-TM), Porto Alegre, Brazil.; 6 Department of Psychiatry and Behavioural Neurosciences McMaster University Hamilton ON Canada Department of Psychiatry and Behavioural Neurosciences, McMaster University, Hamilton, ON, Canada.

Emerging technologies have enabled us to create increasingly accurate predictions about the propensity of psychiatric patients to commit criminal offenses.^1^ Machine learning models raise a variety of opportunities and avenues to develop educational tools, preventive measures, and shape public policy.^2^ However, despite the promise of predictive algorithms in forensic psychiatry, their use raises an important ethical challenge. Namely, how can we avoid further stigmatizing vulnerable individuals, and instead, ensure our algorithms respect their rights, enhance their safety, and promote their wellbeing? The noted philosopher Joel Feinberg envisioned a form of noncomparative justice, where each person is treated precisely as they deserve, without regard to the way anyone else is treated.^3^

To better elucidate this concept, take the example of “voluntary” or “involuntary” criminal acts, which depend on an individual’s intention to commit a crime, otherwise known as *mens rea *(guilty mind)*. *When voluntary criminals are compared against voluntary criminals, such a system is thought to be fair and just in a legal sense. However, when involuntary criminals are compared with voluntary criminals in the same category, and are punished with similar severity, we can discern a state of injustice because of a difference in criminal culpability. As such, the voluntary nature of the criminal act, regardless of the severity of the crime, is a salient consideration.^4^

In many countries, individuals with severe mental illness who commit criminal acts are evaluated according to noncomparative justice.^5^ Rather than simply punishing the offender in proportion to the severity and context of the crime, those with severe mental illness who lack *mens rea* may be treated in a *restorative *framework, recognizing the need to aid, treatment, and seek to prevent future reoffending.^5^ In forensic psychiatry, this implies the need for targeted and individualized treatment.

However, several pertinent questions arise when evaluating the utility and implementation of such algorithms. For instance, an important consideration that is often overlooked is model interpretability. So called “black box” methods may perform well in testing and validation datasets, however, without a rudimentary understanding of the directionality, and interaction effects of important features, we lack the transparency required to justify implementing these models in high stakes clinical settings.^6^ Toward this end, new methods leveraging the internal structure of tree based algorithms can be used to directly measure local feature interaction effects, and provide insight into the magnitude, prevalence, and direction of a feature’s effect.^7^

Similarly, even among classification models that demonstrate high accuracy, there will be instances where individuals are misclassified. In cases where the risks of misclassification are low, this may be largely unimportant. However, when dealing with the complex intersectionality between healthcare, personal freedom, and societal risk, this becomes a challenging consideration. For instance, how can we introduce ethical constraints in our models without significantly impacting their overall accuracy and utility? While this remains open to debate, it may be useful to consider such ethical goals from two distinct frameworks.

Robert Nozick, the renowned American philosopher, once discussed the concept of moral pushes and pulls.^8^ *Moral pushes* involve ideals or values that propel us “from within.” From this framework, ethics are a set of principles that help guide us to being more virtuous individuals. Ethical algorithms can favor these individual moral values if the goal is to make us “better people,” allowing us to live a healthier life, or intrinsically, boosting moral dispositions so that we can better operate within society, leading to the benefit of others by proxy. *Moral pulls*, on the other hand, are constraints about the design of the algorithms. For instance, ensuring that our models are not predicated on immutable characteristics, and ensuring free, informed, and ongoing consent.^8^ The concept of moral pulls also highlights the importance of patient centered perspectives. We argue that a prerequisite for the successful implementation of predictive models into routine care is for data scientists to meaningfully engage with stakeholders (healthcare providers, patients, and their families) to ensure the scope of the problem, and important ethical considerations, are adequately elucidated.

Altogether, we advocate for a marked transformation in the field, where group level statistical approaches to risk assessment, therapeutic interventions, and rehabilitation are abandoned in favor of more precise, individualized models, developed according to a new, precision ethics approach.
